# Identification of *ZHOUPI* Orthologs in Rice Involved in Endosperm Development and Cuticle Formation

**DOI:** 10.3389/fpls.2018.00223

**Published:** 2018-02-28

**Authors:** Mingzhu Dou, Yaohua Zhang, Suxin Yang, Xianzhong Feng

**Affiliations:** ^1^Key Laboratory of Soybean Molecular Design Breeding, Northeast Institute of Geography and Agroecology (CAS), Changchun, China; ^2^College of Life Sciences, Liaocheng University, Liaocheng, China

**Keywords:** rice, transcription factors, *OsZOU-1*, endosperm development, cuticle formation

## Abstract

The endosperm occupies most of the available space within mature rice seeds, contains abundant nutrients, and directly influences both the quality and quantity of rice production. Initial reports noted that AtZHOUPI (AtZOU) coordinates endosperm breakdown and the concomitant separation of the embryo from this structure in *Arabidopsis*. The results of this study show that rice genomes contain two most closely related homologs of *AtZOU*, *OsZOU-1* and *OsZOU-2*; of these, *OsZOU-1* expression is limited to within the endosperm where it can be detected throughout this structure 5 days after pollination (DAP). Its expression gradually decreases from seven DAP to nine DAP. The second of the two most closely related homologs, *OsZOU-2*, is highly expressed in leaves and stem, but is not detected in developing seeds. Heterologous expression of *OsZOU-1* and *OsZOU-2* in *Atzou-4* mutants also revealed that *OsZOU-1* partially complements the seed phenotypes of these individuals, while its counterpart, *OsZOU-2*, was unable to recover these phenotypes. The over-expression of *OsZOU-1* severely disrupts both seed development and plant growth in transgenic rice lines, as plants in which this gene has been knocked down failed in the separation of endosperm from embryo and cuticle formation during seed development. The results of this study therefore suggest that *OsZOU-1* is orthologous to the *AtZOU*, and regulates both endosperm development and cuticle formation in rice.

## Introduction

Angiosperm seeds generally comprise three parts, the embryo, endosperm, and testa. The first of these components, the embryo, is the most important part of the seed as it can develop into the roots, stems, and leaves of plants. In contrast, the endosperm supports the growth and development of the embryo and so performs important roles in nutrient production and transport (Melkus et al., 2009) as well as initiating, transmitting, and integrating inter-compartmental signaling (Weijers and Offringa, 2003; Yang et al., 2008). The third component, the testa, is the ‘armor’ of the seed and performs a key protective role. It is noteworthy that although most angiosperm seeds form endosperm, a number of different modes of development have been recognized; in some mature angiosperm seeds, including soybean and *Arabidopsis*, just one endosperm cell-layer surrounds the embryo (Ingram, 2010) which means that nutrients are absorbed by these structures and stored in cotyledons during development. In contrast, a large endosperm body which will be consumed during germination persists in the mature seeds of monocotyledonous species such as rice and maize, for example (Olsen, 2001).

Rice is a staple food for more than half of the global population (Maclean et al., 2002). Thus, because of the importance of this plant in both agriculture and scientific research, understanding the mechanisms that govern seed growth and development is of considerable significance. Rice endosperm development can be divided into four stages, coenocytic nuclear division, cellularization, the differentiation of an outer aleurone layer and an inner starchy endosperm, and the accumulation of storage products (Brown et al., 1996; Krishnan and Dayanandan, 2003; Olsen, 2004; Becraft and Yi, 2011; Wang et al., 2012). The aleurone layer that comprises one to several cell layers depending on their position accounts for less than 7% of the weight of the rice caryopsis (Juliano, 1993; Becraft and Yi, 2011; Wu et al., 2016). Starchy endosperm cells, in contrast, occupy most of the space within the caryopsis and provide the main source of energy in white rice (Olsen, 2004; Wang et al., 2012; Wu et al., 2016). Programmed cell death (PCD) is one momentous process in the development of endosperm; all of the starchy endosperm cells in mature rice seeds have undergone this process (Olsen, 2004; Kobayashi et al., 2013; Domínguez and Cejudo, 2014), meanwhile PCD and nutrient accumulation in these cells are closely linked and coordinates to one another in time and space (Wang et al., 2012; Kobayashi et al., 2013; Wu et al., 2016).

Initial reports noted that endosperm breakdown during seed development is regulated in *Arabidopsis* by the *ZHOUPI (ZOU)* gene (Kondou et al., 2008; Yang et al., 2008). This gene also regulates the formation of embryonic cuticle and the extra cuticular sheath, as the latter mediates separation of the embryo from the endosperm during seed development in this species (Xing et al., 2013; Moussu et al., 2017). At the same time, AtZOU regulates endosperm breakdown and embryonic cuticle formation via two genetically independent pathways; the first of these involves signaling between the endosperm and embryo (Xing et al., 2013; Denay et al., 2014) as AtZOU triggers PCD within the endosperm by indirectly regulating the expression of enzymes within this structure that function in cell wall modification (Fourquin et al., 2016). The softening of the endosperm cell wall caused by these enzymes appears to be necessary to permit crushing of the endosperm during embryo expansion (Fourquin et al., 2016), while the activation of target gene transcription induced by AtZOU depends on the formation of heterodimers with another bHLH transcriptional factor, INDUCER OF CBF EXPRESSION1 (AtICE1) (Xing et al., 2013; Denay et al., 2014; San-Bento et al., 2014). The ZOU/ICE1 complex participates in intact embryonic cuticle formation by regulating the expression of a subtilisin serine protease, ALE1. The pathway, via which ALE1 promotes the formation of embryonic cuticle, involves receptor kinases GASSHO1 and GASSHO2 expressing in embryo (Xing et al., 2013). Re-introducing ALE1 expression in *zou* mutants therefore partially rescues the formation of embryonic cuticle with persistent endosperm (Xing et al., 2013). The third function of AtZOU is mediating embryo/endosperm separation by promoting the formation of an extra cuticular sheath at the embryo surface. This sheath is deposited outside the embryonic cuticle and incorporates endosperm-derived material that is rich in extensin-like molecules; in this context, the peptide KERBEROS that exhibits ZHOUPI-dependent expression is essential for both embryo sheath formation and embryo-endosperm separation (Moussu et al., 2017). The receptor-like kinases GASSHO1 and GASSHO2 are also required for sheath deposition at the embryo surface but not for the production of sheath material within the endosperm; the normal formation of extra cuticular sheaf and embryonic cuticle is therefore necessary for embryo/endosperm separation as well as to avoid developing organ fusion (Xing et al., 2013; Delude et al., 2016; Moussu et al., 2017).

The *AtZOU* orthologs have been reported in both maize and soybean (Grimault et al., 2015; Zhang et al., 2017), and there is only one *AtZOU* orthologous gene in maize, *ZmZOU*, which expresses within the endosperm. Knock-down of *ZmZOU* retarded the breakdown of both the embryo surrounding region (ESR) and the embryonic suspensor and slightly hindered embryonic growth at early stages. At the same time, the ZmZOU protein forms a range of functional complexes with its AtICE counterpart and partially restores the *Atzou-4* mutant phenotype in endosperm retention and embryo size, as well as cuticle integrity. Nevertheless, the interaction between ZmZOU and the two most closely related homologs of AtICE in maize, ZmICEb and ZmICEc, is weaker than is the case with the structurally divergent and apparently monocot-specific ZmICEa protein. In addition, knock-down of *ZmZOU* does not influence the phenotypes of mature maize kernels or the cuticle integrity of seedlings (Grimault et al., 2015). Two most closely related homologs of *AtZOU*, *GmZOU-1* and *GmZOU-2* are known in soybean where their expression is limited to the seed endosperm. Indeed, when expressed in the *Atzou-4* mutant, just *GmZOU-1* is able to partially complement the mutant phenotype (Zhang et al., 2017).

In order to better understand the function of ZOU orthologs in rice, homologs searches and a phylogenetic analysis were performed in this study which led to the discovery of two most closely related homologs *ZOU* genes. A functional study further demonstrates that *OsZOU-1* shows functional conservation with *AtZOU* and performs important roles in regulating rice endosperm development and cuticle formation even though *OsZOU-2* was unable to rescue the phenotype of the *Atzou-4* mutant. This study presents the first analysis of ZOU function in rice; the results presented in this study will therefore likely prove useful for understanding seed development in cereals.

## Materials and Methods

### Plant Materials and Growth Conditions

The rice cultivar *Nipponbare* (*Oryza sativa* L. ssp. *japonica*) was utilized in this study. Plants were grown under natural conditions in a rice paddy field at Shandong Academy of Agricultural Science, and the *Arabidopsis zou-4* mutant (Col-0 background) that corresponds to GABI_584D09 was obtained from the Gabi-Kat collection of T-DNA inserts (Rosso et al., 2003). The *Arabidopsis* growth method used in this study follows that outlined in previous work (Zhang et al., 2017).

### Phylogenetic Analysis

A total of 211 bHLH protein sequences from rice were mined from Plant Transcription Factor Database V4.0, and a phylogenetic reconstruction of the *AtZOU* gene encompassing rice bHLH proteins was performed by applying the neighbor-joining method (Saitou and Nei, 1987) using the software MEGA7 (Tamura et al., 2007). A bootstrap consensus tree inferred from 1,000 replicates was considered sufficient to represent the evolutionary history of included taxa (Felsenstein, 1985). Branches corresponding to partitions reproduced in less than 50% of bootstrap replicates were collapsed, and the percentage of trees in which associated taxa clustered together (1,000 replicates) were highlighted adjacent to corresponding branches (Felsenstein, 1985). The tree resulting from this analysis was drawn to scale, with branch lengths given using the same units as the evolutionary distances used to infer the phylogeny. Evolutionary distances were computed using the Poisson correction method (Zuckerkandl and Pauling, 1965) and are presented in terms of the number of amino acid substitutions per site. All positions containing alignment gaps and missing data were eliminated via pairwise sequence comparisons (i.e., the pairwise deletion option), an overall total of 671 positions in the final dataset.

### Cloning *OsZOU* Using RT-PCR

Total RNA was isolated from developing rice seeds and leaves under normal growth conditions using the Plant Total RNA Extraction Kit (Bioteke Corporation, Beijing) and first strand complementary DNA (cDNA) was synthesized using the AMV Reverse Transcription System (Takara, Beijing). A 3′ A overhang from EasyTaq (TransGenBiotech) was added to all PCR fragments and these were then cloned into the pMD18-T vector (Takara, Beijing, China). The primers OL0069 and OL0070 were used for *OsZOU-1* clone, while OL0071 and OL0072 were used for *OsZOU-2* clone (Supplementary Table S1).

### Quantitative RT-PCR (qRT-PCR) Expression Analysis of *OsZOU*

Rice total RNA extraction, reverse transcription, and qRT-PCR were all performed following previously described protocols (Dou et al., 2017), as was *Arabidopsis* RNA extraction (Zhang et al., 2017). The reference genes *Actin1* (rice) and *EIF4* (*Arabidopsis*) were used for expression level calculations (Xing et al., 2013; Dou et al., 2017) in all cases and all primers are listed in Supplementary Table S1.

In order to analyze *OsZOU* expression patterns in rice, total RNA was isolated from a range of different tissues, including root, stem, leaf, unopened flower, and kernels at between two and nine DAP. Similarly, to analyze *OsZOU-1* expression levels in different rice seed segments, the endosperm and embryo were obtained separately from rice kernels at five DAP following the protocol outlined by Zhang et al. (2017). Error bars denote the standard deviation (SD) calculated using three technical replicates on pooled material from three WT plants.

In order to analyze *OsZOU* expression levels in *Atzou-4* mutants carrying the *pAtZOU::OsZOU-1* or *pAtZOU::OsZOU-2* constructs, five independent transgenic lines were examined in each case. Three different plants from each line were used for biological replicates and the *Atzou-4* mutant was selected as a negative control. Three consecutive siliques from the mid to late heart stage of each plant were picked and flash frozen in liquid nitrogen for RNA extraction (Xing et al., 2013). *OsZOU* expression was then quantified relative to the *AtZOU* level in Col-0 seeds; the error bars in these cases represent SDs calculated from three biological replicates (Student’s *t*-test value: ^∗∗^*P* < 0.01).

To analyze the expression levels of *OsZOU-1* in the *OsZOU-1* knock-down transgenic lines, the seeds at five DAP of WT line and knock-down lines KDZ-2, KDZ-4 were examined by qRT-PCR. Three homozygous plants from each line were examined, and the expression values are averages for three plants. Thus, *OsZOU-1* expression level in WT seeds was set as 1 and the error bars in this case correspond to the SD calculated from three biological replicates (Student’s *t*-test value: ^∗∗^*P* < 0.01).

To analyze *OsZOU-1* expression levels in *OsZOU-1* over-expressed transgenic lines, unopened flowers of OXZ-4, OXZ-5, and WT plants were picked, flash frozen in liquid nitrogen, and used for RNA extraction and qRT-PCR. The *OsZOU-1* expression level in WT plants was again set as 1, and error bars in this case correspond to the SD calculated from four technical replicates on pools of eight unopened flowers from WT, OXZ-4, and OXZ-5 individuals, respectively (Student’s *t*-test value: ^∗∗^*P* < 0.01).

### Vector Construction and *Arabidopsis* Transformation

In order to construct *pAtZOU::OsZOU-1* and *pAtZOU::OsZOU-2* expression vectors, cloned *OsZOU-1* and *OsZOU-2* cDNA from pMD18-T vectors were digested with *Xba*I and *Hin*cII and cloned into the pRT101 vector. This enabled construction of the vectors ZYH040 and ZYH041 which were then digested with *Sal*I and *Hind*III before gene fragments were separately cloned into a binary vector pCAMBIA1301 to construct ZYH057 and ZYH058. A 1.6-kb region upstream from the *AtZOU* start codon in pSC-B (Zhang et al., 2017) was then digested with *Sal*I, and the promoter fragment was inserted into the binary vectors ZYH057 and ZYH058 to construct the *pAtZOU::OsZOU-1* and *pAtZOU::OsZOU-*2 expression vectors ZYH065 and ZYH067. All recombinant plasmids were verified via sequencing, and the correct ones were introduced into *Agrobacterium tumefaciens* GV3101 independently for plant transformation. All *Arabidopsis* transformations were performed following previously described protocols (Zhang et al., 2017).

### Selection of Transformed *Arabidopsis* Plants

Homozygous transgenic *Arabidopsis* plants were screened using previously developed protocols (Zhang et al., 2017), and transgenic lines were further verified for the proper T-DNA insertion containing the exogenous *OsZOU-1* and *OsZOU-2* derived by the *AtZOU* promoter. The *Atzou-4* mutant background in obtained *OsZOU-1* and *OsZOU-2* transgenic lines was identified using PCR based on the presence of a relevant T-DNA insertion site.

### Toluidine Blue (TB) Staining

A series of TB staining tests were performed on *Arabidopsis* seedlings following protocols developed in previous work (Tanaka et al., 2004; Zhang et al., 2017); tests were therefore performed independently on *OsZOU-1#1*, *#4*, and *#5* and *OsZOU-2#1*, *#3*, and *#5 Arabidopsis* transgenic plants. To do this, a total of 50 *Arabidopsis* seeds from each plant were grown uniformly on 15 cm plates; these were then dyed and photographed and 20 seedlings from each plant line were harvested in order to quantify their TB absorption values. Roots and seed coats (both of which stain dark when treated with TB) were completely removed before the cotyledons and hypocotyl were incubated with 80% ethanol and subjected to spectrophotometer analysis. Mean values for *OsZOU-1#1*, *#4*, and *#5* represent results for *OsZOU-1* transgenic lines, while those for *OsZOU-2#1*, *#3*, and *#5* represent results for *OsZOU-2* transgenic lines. Error bars in these cases denote SD values for three plants (Student’s *t*-test value: ^∗∗^*P* < 0.01, ^∗^*P* < 0.05).

Three homozygous plants from the knock-down rice lines KDZ-2 and KDZ-4 were used for TB staining analyses in each case. Six seeds from each plant were placed in plates containing tap water in a greenhouse (subject to cycles of 14 h light and 10 h dark at 28°C) for germination. This water was changed once a day to keep it clean, and rice seedlings were placed in plates containing staining solution [i.e., 0.4% (v/v) Tween-20 and 0.05% (w/v) TB] for 2 min on the third day after germination. Seedlings were then washed under running tap water until no visible blue color remained before they were photographed.

### Vector Construction and Rice Transformation

The *OsZOU-1* cDNA from the pMD18-T vector was digested with *Eco*RI and *Hinc*II and connected into a pRT101 vector that had been digested using *Eco*RI and *Sma*I. This enabled the ZYH070 construction which was then digested using *Hind*III and *Eco*RI before a DNA fragment was connected into the binary vector pCAMBIA1300 to construct ZYH071. A 1.9 kb region upstream from the *OsZOU-1* start codon was then amplified using PCR with the primers ZOUECORIF and ZOUECORIR and subcloned into vector pMD18-T. The vector containing the *OsZOU-1* promoter was then digested with *Eco*RI, and the promoter fragment was connected into the binary vector ZYH071 to construct the *pOsZOU-1::OsZOU-1^AS^* expression vector ZYH074. Similarly, to construct a *pOsZOU-1::GUS* expression vector, ZYH076 containing the *OsZOU-1* promoter was digested with *Pst*I and *Bam*HI and connected into the binary vector *pCXGUS-P* (Chen et al., 2009). This process enabled construction of the GUS report vector ZYH081, while to construct a *OsZOU-1* over-expression vector, *OsZOU-1* was amplified using PrimeSTAR HS DNA Polymerase (Takara, Beijing, China) and the primers OL0069 and OL0070. A series of 3′ A overhangs were then connected to PCR fragments using EasyTaq (TransGenBiotech, Beijing, China) and the products were directly connected into the binary vector pCXUN (Chen et al., 2009) by T-A cloning enabling construction of the *OsZOU-1* over-expression vector ZYH048. All of these vectors were then transformed into the *Nipponbare* (*Oryza sativa* L. ssp. *japonica*) rice cultivar using the *Agrobacterium*-mediated co-cultivation method (Hiei et al., 1994). Hygromycin-resistant plants were then screened for the presence of the transgene via PCR using gene specific primers, and seeds from different transgenic individuals were harvested separately. Primary transformants were referred to as T1 transgenic plants, and homozygous individuals were screened using an MS medium containing hygromycin B; T3 homozygous knock-down transgenic individuals were used for subsequent phenotypic and molecular analyses.

### GUS Staining

GUS report rice kernels at five DAP were cut into longitudinal sections with a razor blade and placed in pre-cooled 90% acetone for 20 min. These kernels were then rinsed twice with a X-Gluc free GUS staining solution, following a previously developed procedure (Maharjan et al., 2011).

### Cell Numbers and Size Measurements

Cell numbers and size measurements for *OsZOU-1* over-expressed lines and WT lines were recorded based on ten seeds of each plant via photographs taken with a Nikon Eclipse E800 microscope. Cell numbers and lengths at the widest point of glumes in each case were measured using the software Image J. Error bars in this case correspond to SDs calculated from all measurements (Student’s *t*-test value: ^∗∗^*P* < 0.01).

## Results

### Isolation and Cloning of *OsZOUs*

**FIGURE 1:**
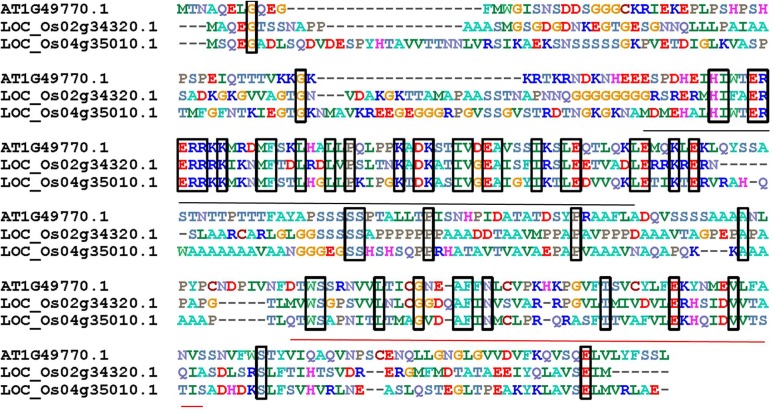
The alignment of *AtZOU* and two most closely related rice homologs. Alignment of amino acid sequences of *AtZOU* (AT1G49770.1) with *OsZOU-1* (LOC_Os04g35010) and *OsZOU-2* (LOC_Os02g34320) genes. Identical amino acids shared between these three proteins are framed in black rectangles; the bHLH region is indicated by a black line below the alignment, while the conserved region at the C-termini of proteins is indicated with a red line below the alignment.

In order to identify *ZOU* orthologous genes, a AtZOU amino acid sequence was used as a bait to search for orthologs within the rice genome (Phytozome). Results show that two *ZOU* homologs (*LOC_Os04g35010* and *LOC_Os02g34320*) were highly conserved with *AtZOU* and the corresponding genes were termed *OsZOU-1* and *OsZOU-2*, respectively. Amino acid sequences alignment of *OsZOUs* with *AtZOU* revealed conservation within the bHLH DNA-binding domain (pfam00010) and C-terminal region (**Figure 1**). Phylogenetic analysis of all the 211 bHLH family proteins in *Oryza sativa subsp. japonica* with AtZOU revealed that OsZOU-1 and OsZOU-2 were located within the same clade with AtZOU, while the other bHLH family proteins were not (Supplementary Figure S1).

### *OsZOU* Expression Patterns

**FIGURE 2:**
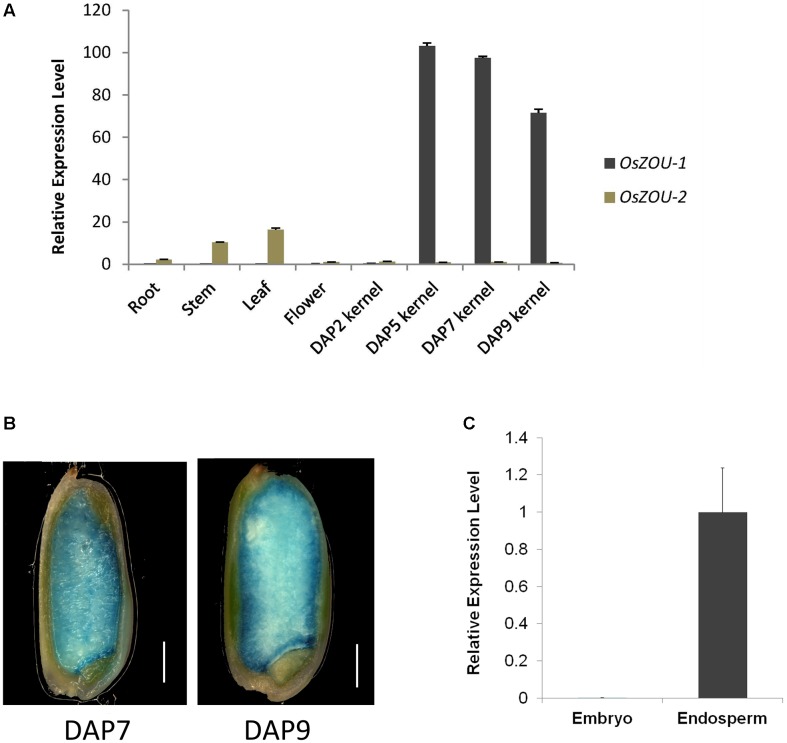
*OsZOU-1* and *OsZOU-2* expression analyses. **(A)** Expression of *OsZOUs* in different tissues, including the root, stem, leaf, unopened flower, and seeds at different DAPs, as detected by qPCR analysis. The expression level of *OsZOU-2* in unopened flowers was set as 1, and all experiments were repeated three times. **(B)** GUS staining of *pOsZOU-1::GUS* transgenic line seeds at seven DAP and nine DAP. Scale bars = 1 mm. **(C)** qPCR analysis of *OsZOU-1* expression in dissected seed compartments. The embryo and endosperm were isolated separately from developing seeds at five DAP, and the expression level of *OsZOU-1* in the endosperm was set as 1. Error bars in **(A,C)** denote SDs calculated from technical triplicates on pools of material from three plants, with *AtEIF4* used as the reference gene.

A series of qPCR experiments were performed using RNA extracted from vegetative and reproductive tissues, including roots, stems, leaves, unopened flowers, and developing kernels at different stages to analyze tissue specific expression of *OsZOUs* (**Figure 2A**). These results reveal that *OsZOU-1* is mainly expressed in kernels between five and nine DAP and that there is a peak at five DAP when endosperm cellularization has completed and no visible expression of *OsZOU-1* can be detected in roots, leaves, and stems. In contrast, data show that *OsZOU-2* is mainly expressed in leaves and stems, at much lower levels in roots, and is almost completely absent from kernels (**Figure 2A**).

To further dissect the promoter activity of *OsZOU-1* in developing seeds, twenty-three independent *pOsZOU-1::GUS* transgenic plant were developed in which 1.9 kb of *OsZOU-1* promoter drived GUS coding sequence. Seeds at seven DAP and nine DAP were stained with a GUS solution and visualized under microscope (**Figure 2B**); these results reveal a GUS signal was detected in entire endosperm of kernels at seven DAP and nine DAP but not in embryo. Thus, to further determine the expression position of *OsZOU-1* in developing rice kernels, the relative expression levels of *OsZOU-1* in dissected embryos and endosperms at five DAP were examined. The qPCR results reveal that *OsZOU-1* is highly expressed in the endosperm, but not in the embryo (**Figure 2C**). These results show that *OsZOU-1* is specifically expressed in developing kernel endosperm but not in the embryo.

### Heterologous Expression of *OsZOU-1* Rescued *Atzou* Mutant Phenotype

**FIGURE 3:**
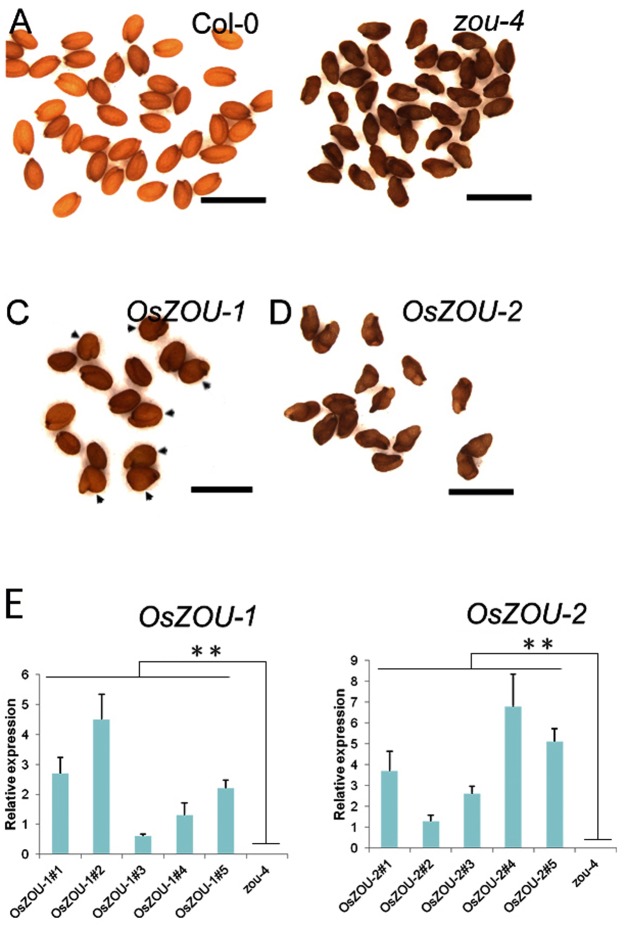
Heterologous expression of *OsZOUs* in the *Atzou-4* mutant. **(A–D)** Seed phenotypes of Col-0, *Atzou-4*, *OsZOU-1*, and *OsZOU-2* transgenic lines (the seed phenotypes of independent transgenic events *OsZOU-1#1*, *#4*, *#5* and *OsZOU-2#1*, *#3*, *#5* were observed, but only images from *OsZOU-1#4* and *OsZOU-2#3*, considered representative for all other events, are presented here). The seeds from Col-0 are full **(A)** while those from *zou-4* are abnormally shriveled **(B)**. In *OsZOU-1* transgenic lines, the seeds are plump and some show irregular shapes, indicated by black arrow heads **(C)**, while the seeds of *OsZOU-2* transgenic lines were shriveled, similar to the *Atzou-4* mutant **(D)**. Scale bars = 1 mm. **(E)** qPCR analyses were performed to analyze the expression levels of *OsZOUs* in transgenic seeds at the heart stage. Five independent transgenic lines from each were examined and the un-transformed *Atzou-4* mutants were selected as a negative control. The expression of *OsZOUs* was quantified relative to *AtZOU* expression in Col-0 seeds. Error bars represent the standard deviation calculated from three biological replicates, each containing three consecutive siliques at the mid to late heart stage, with *AtEIF4* used as the reference gene. Student’s *t*-test value: ^∗∗^*p* < 0.01.

In order to investigate whether, or not, *OsZOUs* are involved in endosperm breakdown, *Atzou-4* mutants were transformed with *OsZOU-1* and *OsZOU-2* controlled by the *AtZOU* promoter (*pAtZOU::OsZOUs*), respectively (**Figure 3**). Five independent transgenic lines of *OsZOU-1* and *OsZOU-2* were tested separately in this experiment and *OsZOU-1* and *OsZOU-2* were successfully expressed in seeds in all cases (**Figure 3E**). Phenotype analyses were therefore performed on three transgenic lines in which *OsZOU-1* or *OsZOU-2* were moderately expressed. Observation revealed that *OsZOU-1* almost completely rescued the shriveled seed phenotype and that the mature dry seeds from *OsZOU-1* transformation lines remained plump albeit irregularly shaped in some cases (**Figures 3A,C**). Mature seeds from *OsZOU-2* transgenic lines were shriveled, similar to their counterparts from *Atzou-4* mutant lines (**Figures 3B,D**).

**FIGURE 4:**
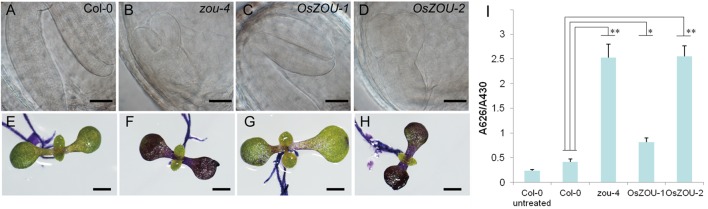
Seed development and embryonic cuticle integrity. Phenotype analyses of the independent transgenic events *OsZOU-1#1*, *#4*, and *#5* and *OsZOU-2#1*, *#3*, and *#5* were performed, but only images from *OsZOU-1#4* and *OsZOU-2#3*, considered representative of all other events, are presented here. **(A–D)** The embryo of Col-0, *Atzou-4*, *OsZOU-1* transgenic, and *OsZOU-*2 transgenic plants at ten DAP were observed under Differential Interference Contrast (DIC) microscopy. Scale bars = 0.1 mm. The embryo of Col-0 plant was bent, almost occupying the entire seed cavity with only one cell layer of endosperm surrounding the embryo **(A)**. The *Atzou-4* mutant embryo was arrested in the heart/torpedo stage and its endosperm remained intact **(B)**. In *OsZOU-1* transgenic seeds, the bent embryo occupied most of the seed cavity, and the endosperm showed breakdown **(C)**. The embryo and endosperm of *OsZOU-2* transgenic seeds were the same as those of the *Atzou-4* mutant **(D)**. Scale bars in **(A–D)** are 50 μm. **(E–I)** *OsZOU-1* partially recovered the cuticle deficiency in *Atzou-4*. Scale bars in **(E–I)** are 1 mm. Seedlings of the Col-0 **(E)**, *Atzou-4* **(F)**, *OsZOU-1* **(G)**, and *OsZOU-2* **(H)** transgenic lines on the seventh day after germination were stained with TB. Results show that *OsZOU-1* transgenic seedlings were weakly stained **(G)**, while those of the *OsZOU-2* seedlings showed strong staining similar to that in the *Atzou-4* mutants **(H)**. TB uptake in these lines was quantified spectrophotometrically and revealed similar results to microscopy analysis. The mean values of *OsZOU-1#1*, *#4*, and *#5* represent the results for *OsZOU-1* transgenic lines, while the mean values of *OsZOU-2#1*, *#3*, and *#5* represent the results for *OsZOU-2* transgenic lines. Error bars denote the SDs of three biological replicates, each containing 20 seedlings **(I)**. Student’s *t*-test value: ^∗^*p* < 0.05, ^∗∗^*p* < 0.01.

Developing seeds from transgenic lines were examined in order to verify the function of *OsZOUs* during embryonic and endosperm growth (**Figures 4A–D**). Results showed that the Col-0 embryo was almost completely bent with little endosperm retention (**Figure 4A**), while the embryo of the *Atzou-4* mutant was hindered at the heart/torpedo stage and a large volume of endosperm persisted, which resulted from PCD failure (**Figure 4B**). The embryos from the *OsZOU-1* transgenic line were bent and occupied most of the embryonic sac, similar to Col-0, meanwhile the endosperms from the *OsZOU-1* transgenic lines exhibited degradation with less persistent compared to *Atzou-4* mutants (**Figure 4C**). In contrast, the seeds from the transgenic lines with over-expressed *OsZOU-2* were similar in their development to the *Atzou-4* mutant as embryo morphology was arrested in the heart/torpedo stage and a large volume of endosperm was preserved (**Figure 4D**). The results of this study indicate that *OsZOU-1* rescues the defect in endosperm breakdown and embryo expansion partly, although *OsZOU-2* is unable to recover seed development in *Atzou-4* mutants.

The integrity of embryonic cuticle in transgenic lines was also examined in this study because of breaks observed in *Atzou* mutants. As discussed, hydrophobic cuticle defects were detected using the TB hydrophilic dye (Tanaka et al., 2004); thus, on the seventh day after germination, seedlings of *zou-4* mutants, Col-0 WT plants, and *OsZOU* transgenic lines were dyed with a TB aqueous solution and imaged using a microscope. Observations reveal that the cotyledons of *zou-4* lines were strongly stained, while the dye was hardly able to penetrate this region in Col-0 WT plants. Staining results for the cotyledons of *OsZOU-2* transgenic plants were similar to those of *zou-4* mutants, while *OsZOU-1* transgenic individuals were only weakly dyed (**Figures 4E–H**). Finally, the TB absorption capacity of seedlings was also quantified using spectrophotometry, yielding results consistent with microscope observations (**Figure 4I**). These findings indicated heterologous expression of *OsZOU-1*, but not *OsZOU-2* also partially rescued cuticle formation in *Atzou-4* mutant.

### Knock-down of *OsZOU-1* Influences Endosperm Development and Cuticle Formation in Rice

**FIGURE 5:**
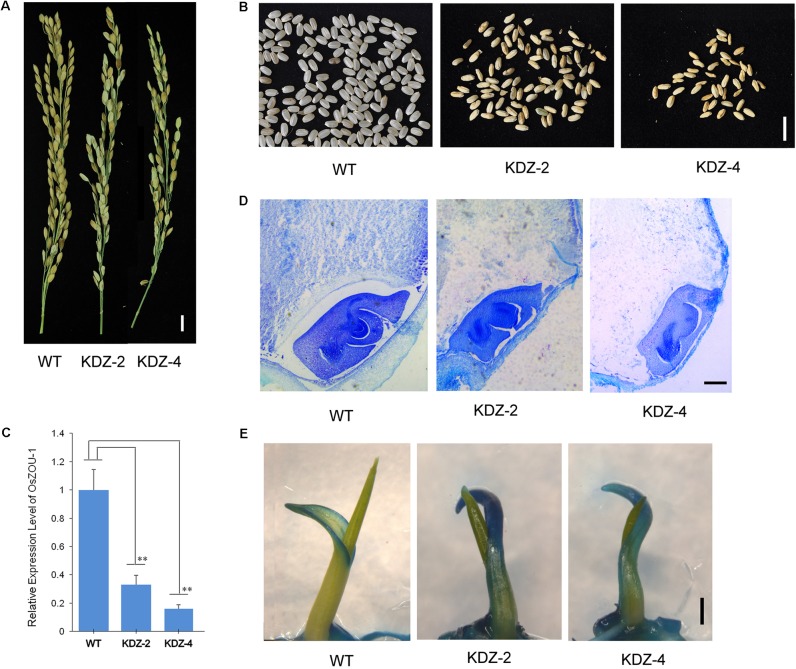
Phenotype of *OsZOU-1* knock-down lines. Three homozygous plants from the knock-down lines KDZ-2 and KDZ-4 were used for phenotype and molecular analyses, but only one image from each is presented here. **(A)** Spikes of *OsZOU-1* knock-down transgenic lines with fewer full seeds compared to the WT line. Scale bar = 1cm. **(B)** Mature seeds from the WT line and the knock-down lines KDZ-2 and KDZ-4. Scale bar = 1 cm. **(C)** Relative expression levels of *OsZOU-1* in WT seeds five DAP and in the knock-down lines KDZ-2 and KDZ-4 were examined using qRT-PCR analysis. Expression values for each line are averages for three plants; the expression level of *OsZOU-1* in WT seeds was set as 1. Error bars correspond to the SD calculated from three biological replicates, and *OsActin1* was used as the reference gene. Student’s *t*-test value: ^∗∗^*p* < 0.01. **(D)** Cross sections of WT and KDZ seeds at 12 DAP. Scale bars = 100 μm. **(E)** WT seedlings, KDZ-2, and KDZ-4 on the third day after germination were stained with TB. Just the margin of the coleoptile was weakly stained in WT seedlings, while the whole coleoptile in KDZ-2 and KDZ-4 was strongly stained. Scale bars = 1 mm.

In order to further investigate the function of *OsZOU-1*, a series of knock-down plants were also generated as part of this study. Thus, *OsZOU-1* knock-down lines were generated via expression of full length antisense *OsZOU-1* cDNA sequences under the control of a 1.9 kb *OsZOU-1* endogenous promoter. A total of 63 independent T1 transformation plants were generated and T-DNA insertions were verified by PCR. Observations show that these transgenic plants exhibited no visible defects with the exception of lower seed rates than their WT counterparts (**Figure 5A** and Supplementary Figures S3, S5); meanwhile, most mature transgenic kernels were not completely filled (**Figure 5B**) with the full seed percentages less than 20% in the ten primary transgenic plants caculated (Supplementary Figure S4). Data show that *OsZOU-1* expression levels in the T1 knock-down plants KDZ-2 and KDZ-4 were about 33 and 16% of the WT kernel level at five DAP, respectively (**Figure 5C**); histological analysis also revealed that embryos remained adhering to the surrounding endosperm in knock-down kernels at 12 DAP, whereas there was endosperm cavity between endosperm and embryo in wild type kernel (**Figure 5D**).

The integrity of embryonic cuticle in knock-down rice lines was examined using a TB staining test. Thus, seedlings of WT, KDZ-2, and KDZ-4 individuals were stained with a TB solution and observed on the third day after germination. Results show that just coleoptile margins were weakly stained in WT seedlings, while the whole of this structure in KDZ-2 and KDZ-4 plants were strongly stained (**Figure 5E** and Supplementary Figure S6). This result suggests that the rice seed cuticle becomes defective when *OsZOU-1* expression is knocked down.

### Over-expression of *OsZOU-1* Affects Rice Plant Development

**FIGURE 6:**
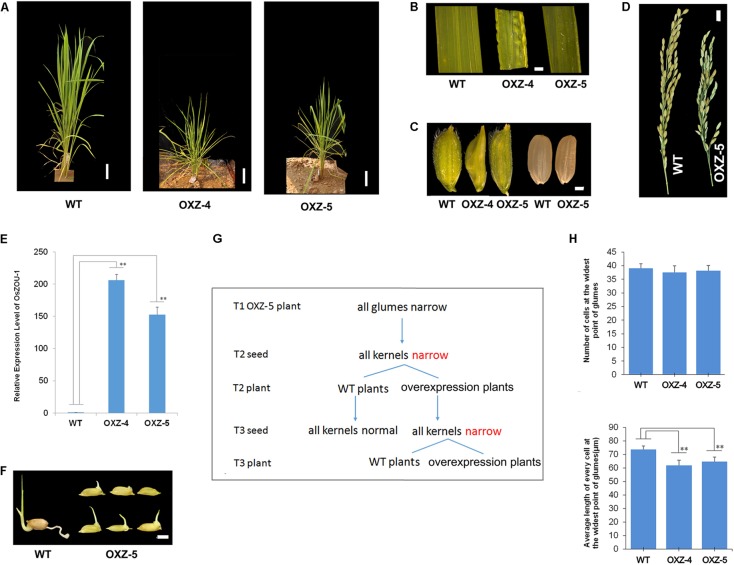
The phenotype of *OsZOU-1* over-expression lines. **(A)** The morphology of WT and *OsZOU-1* over-expressed plants OXZ-5 and OXZ-4 in the jointing-booting stage were photographed. Scale bars = 8 cm. The OXZ-5 plant (54.48 cm) and OXZ-4 plant (47.52 cm) were shorter than the WT plant (81.28 cm). **(B)** The leaves of OXZ-5 and OXZ-4 curled upward along the leaf margin before booting and were narrow, while some leaves of OXZ-4 were uneven. Following booting, the upward curling leaf blade phenotype changed. **(C)** The glumes and kernels of OXZ-5 and OXZ-4 were narrower than WT. **(D)** Spikes of WT and OXZ-5 plant at the mature stage. Scale bar = 1 cm. The over-expression of *OsZOU-1* led to reduced fertility. **(E)** The relative expression levels of *OsZOU-1* in unopened flowers of WT and transgenic lines. *OsActin1* was used as the internal control and the expression level of *OsZOU-1* in unopened flowers of WT plant was set as 1. The expression levels of *OsZOU-1* in unopened flowers of OXZ-4 and OXZ-5 were respectively 206 and 152 times higher than WT. Error bars correspond to the SDs calculated from four technical replicates on pools of eight unopened flowers from the WT, OXZ-4, or OXZ-5 plants, respectively. **(F)** The germination situation of WT and OXZ-5 seeds that were put in water six days previously. These results show that the germination of OXZ-5 seeds was difficult and inconsistent. Scale bar = 0.5 cm. **(G)** Heritability of the narrow kernel phenotype in OXZ-5 transgenic plants. **(H)** Cell numbers and lengths at the widest point of the glumes of WT and over-expressed plants. Results are means ± SD (*n* = 10); Student’s *t*-test value: ^∗∗^*p* < 0.01.

A series of over-expressed transgenic lines, in which *OsZOU-1* cDNA was driven by a maize ubiquitin promoter for efficient expression in monocots (Chen et al., 2009),were generated to explore *OsZOU-1* function in rice. Although seven independent transgenic plants were generated, their phenotypes were similar and so just two, OXZ-4 and OXZ-5, were selected for further analysis. These plants exhibited dwarfism and leaf curling, had narrowed leaves, glumes, and kernels, and poor fertility (**Figures 6A–E** and Supplementary Figures S7, S8). The expression of *OsZOU-1* in unopened OXZ-4 flowers was 206 times the level in WT plants, and in unopened OXZ-5 flowers was 152 times the level in WT plants (**Figure 6E**). At the same time, OXZ-4 plant was infertile and more dwarfed than their OXZ-5 counterpart (**Figure 6A** and Supplementary Figure S7), which exhibited the consistency of the phenotypic severity with the expression levels. Although the OXZ-5 plant produced fertile seeds, germination was difficult and inconsistent (**Figure 6F** and Supplementary Figure S9).

The results of this study also show that the narrow kernel phenotype is inherited maternally. All the glumes and kernels of over-expressed plants were narrow in this study; these narrow kernels developed into both over-expressed and WT plants that had normal glumes and kernels (**Figure 6G**). This means that kernel width is controlled by the glume, a component of the female parent, and not by the genotype. Cell number and length along the widest part of the glume was therefore measured in order to explore the direct cause of this narrow phenotype; data show that the glume cell numbers in over-expressed plants exhibited no obvious differences when compared to the WT although the cell length of glumes in OXZ-4 (61.89 μm) and OXZ-5 (64.78 μm) were both significantly smaller than in the WT (73.85 μm) (**Figure 6H**). These results show that the correct expression of *OsZOU-1* is important for both rice seed and plant development.

## Discussion

### *OsZOU-1* Is the Rice Ortholog of *AtZOU* With Conserved Function

Although *ZOU* has previously been shown to be important in endosperm breakdown and cuticle formation during *Arabidopsis* seed development (Wei et al., 2002; Yang et al., 2008; Xing et al., 2013; Denay et al., 2014), its function in rice seed development remains poorly understood. The results of this study show that although there are two most closely related homolog of *AtZOU* in rice, just *OsZOU-1* complements the defects of cuticle integrity, endosperm breakdown, and embryo expansion in the *Atzou-4* mutant, whereas *OsZOU-2* failed to rescue *Atzou-4*. In *OsZOU-1* knock-down lines, the endosperm cavity did not form at 12 DAP with embryo adhering to the endosperm, which might be caused by the PCD failure in the endosperm adjacent to the embryo since endosperm cavity appears as soon as the endosperm adjacent to the embryo has degraded in maize and wheat (Young and Gallie, 2000; Cosségal et al., 2007). This result implies that *OsZOU-1* shows functional conservation with *AtZOU*.

### Conservation of the *ZOU* Gene Throughout Plant Evolution

The *ZOU* gene is found ubiquitously from mosses to seed plants, its presumed ancestral function is promoting the separation of the embryo from surrounding gametophytic tissues, as well as their subsequent breakdown (Yang et al., 2008). To date, *ZOU* orthologs have been identified in *Arabidopsis*, soybean, maize, and rice; interestingly, just only one copy of *ZOU* in each species can complement the *Arabidopsis zou* mutant phenotype, even though in soybean with duplicated genome. This may imply that although the *ZOU* gene performs important and conserved roles during endosperm development, a number of duplicated genes have diverged and lost this original function because of its highly specific expression pattern.

The phylogenetic analysis presented in this paper also suggests that *ZOU* genes in monocotyledonous plant species can be divided into three subgroups (Supplementary Figure S2). In this context, it is interesting to note that *ZOU* orthologous genes that complement the *zou* mutant phenotype, such as *OsZOU-1* and *ZmZOU*, occur in clade1 while their corresponding homologs generated by genomic duplication or gene duplication all occur in clade3. Similarly, the *ZOU* genes that are duplicated in *Brachypodium distachyon* (*Bradi*) and *B. stacei* (*Brast*) occur within clade2; whether, or not, these perform a similar role to their counterparts in clade1 currently remains unclear. Since *OsZOU-2* was highly expressed in vegetative tissues, and cannot complement the *zou* mutant phenotype, it can be hypothesized that the *ZOU* orthologousgenes in clade3 may have lost the original function of this gene via changes of their expression patterns or protein sequence conservation.

Differentiation in endosperm development between dicots and monocots confirms that research on *ZOU* in species of the latter is valuable for exploring its functional conservation or variation associated with endosperm evolution. As a result, further experiments are needed to explore the role of *ZOU* in regulating endosperm development in rice, and comparisons of the role of *ZOU* in monocotyledon species versus certain dicotyledonous ones that possess non-degraded endosperm are also useful in investigating the evolution of endosperm development process.

## Conclusion

Two most closely related homologs of *AtZOU*, *OsZOU-1* and *OsZOU-2*, are identified in this study; results show that the first of these is expressed in rice endosperm while the second is expressed to a high level in both leaves and stems. Data show that the heterologous expression of *OsZOU-1* partially complements the seed phenotype of the *Atzou-4* mutant, while *OsZOU-2* was unable to recover this mutant phenotype. The over-expression and knock-down of *OsZOU-1* disrupted rice seed development. The results of this study present one possible mechanism that might underlie endosperm development and cuticle formation during the growth of rice seeds.

## Author Contributions

SY and XF designed the research. YZ cloned the *OsZOU* genes and performed the phylogenetic analysis and mutant complementation. MD carried out the transgenic study, gene expression, cell-level and other functional analyses. XF, SY, MD, and YZ wrote the paper.

## Conflict of Interest Statement

The authors declare that the research was conducted in the absence of any commercial or financial relationships that could be construed as a potential conflict of interest.
