# Supplementary material for: Identification of ZHOUPI Orthologs in Rice Involved in Endosperm Development and Cuticle Formation
Source: Front Plant Sci. 2018 Feb 28;9:223. doi: 10.3389/fpls.2018.00223 (PMC5835929; doi:10.3389/fpls.2018.00223)
Supplement: Supplementary file 1 [file Presentation_1.pdf]

Identification of *ZHOUPI* Orthologs in Rice Involved in Endosperm Development and Cuticle Formation

Mingzhu Dou[1,2#], Yaohua Zhang[1#], Suxin Yang[1]*, and Xianzhong Feng[1]*

1 Key Laboratory of Soybean Molecular Design Breeding, Northeast Institute of Geography and Agroecology, Chinese Academy of Sciences, Changchun, China

2 College of Life Sciences, Liaocheng University, Liaocheng, China

[#]These authors contributed equally to this work.

*Correspondence:

Suxin Yang

E-mail: yangsuxin@iga.ac.cn

Tel. 0086-431-88542365

Fax 0086-431-85542298

OR

Xianzhong Feng

E-mail: fengxianzhong@iga.ac.cn

Tel. 0086-431-85655051

Fax 0086-431-85542298

Supplemental material

Supplementary Figure S1 Phylogenetic analysis of the bHLH family proteins in rice with AtZOU.

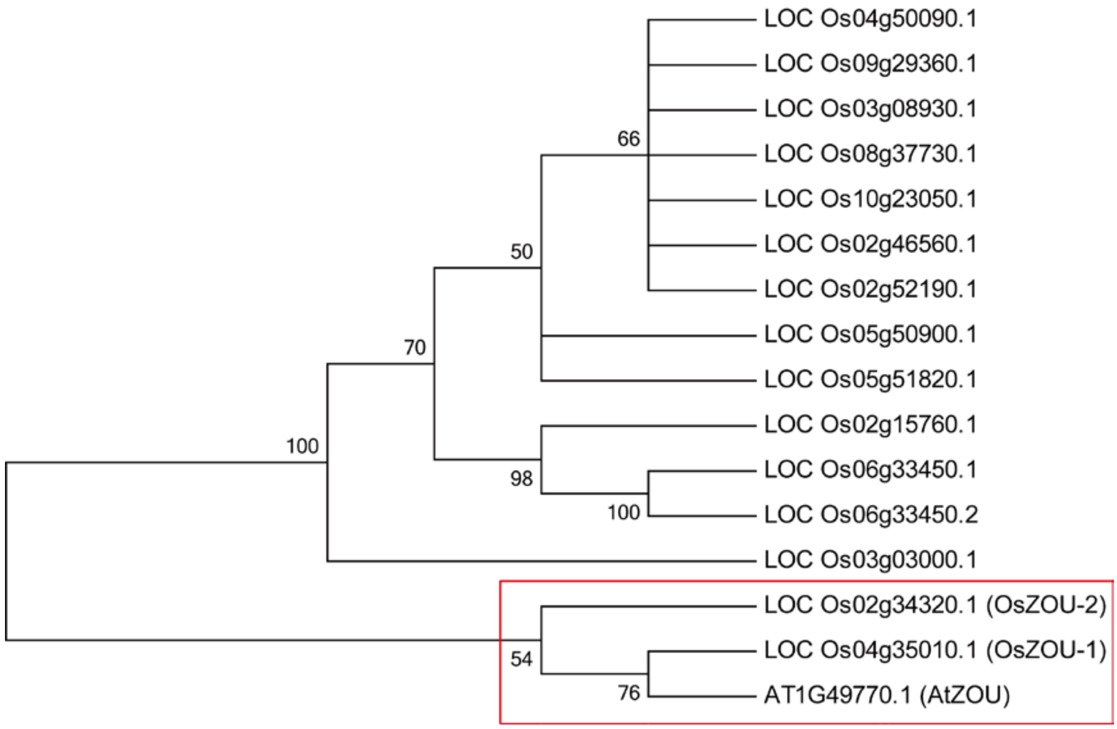

Supplementary Figure S2 Evolutionary relationships of ZOUs in monocotyledon species. The phylogenetic analysis of ZOUs in representative members of monocot species, including *oryza sativa* (Os), *Zea mays* (Zm), *Oropetium thomaeum* (Oropetium), *Brachypodium distachyon* (Bradi), *Brachypodium stacei* (Brast), *Sorghum bicolor* (Sobic), *Setaria italic* (Seita) and *Panicum virgatum* (Pavir). These genes can be divided into three groups based on differences among their full-length protein sequences and the relationship of homologs in certain species. The *Arabidopsis* bHLH protein At5g43650 serves as an outgroup.

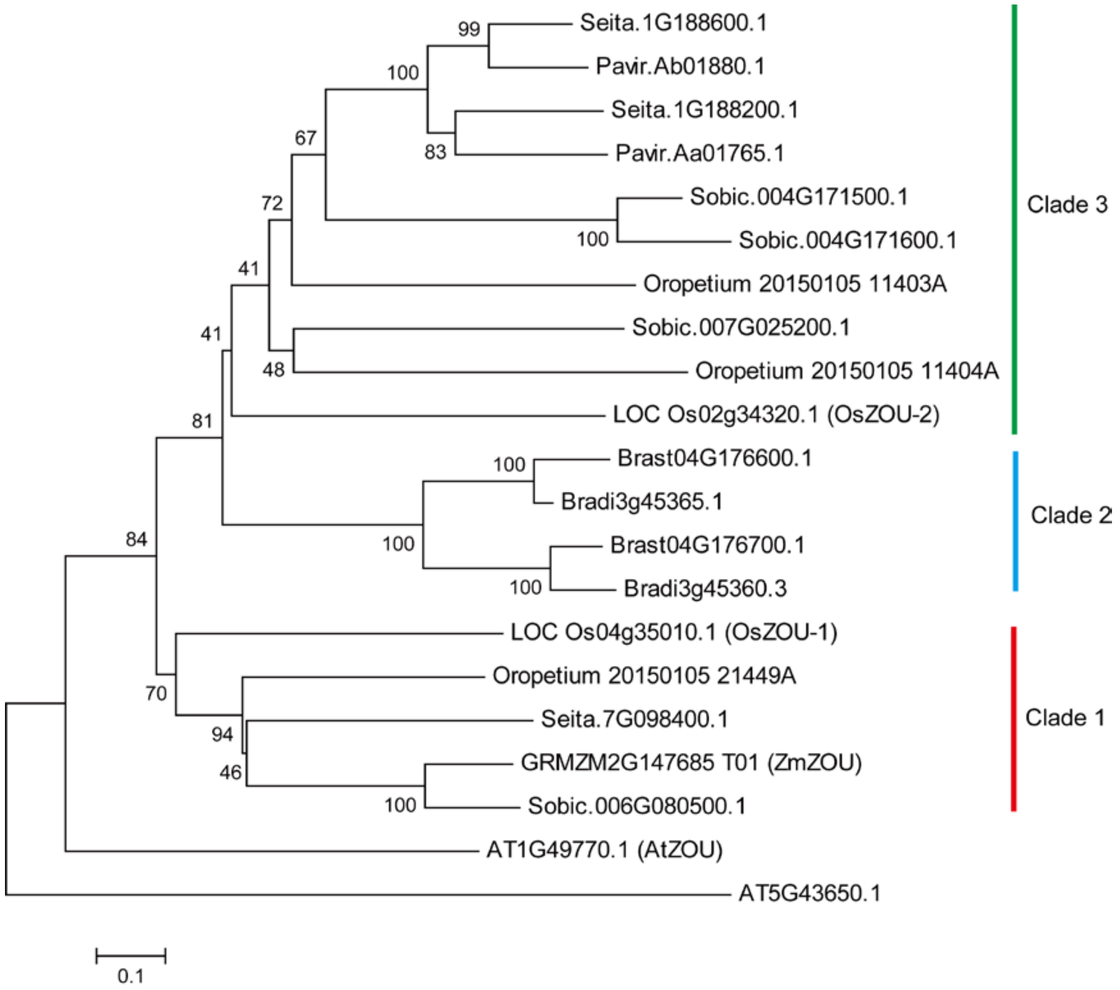

Supplementary Figure S3 Seed setting rates of primary *OsZOU-1* knock-down plants. The total number of seeds divided by the total number of spikelets represents the seed setting rate of the plant.

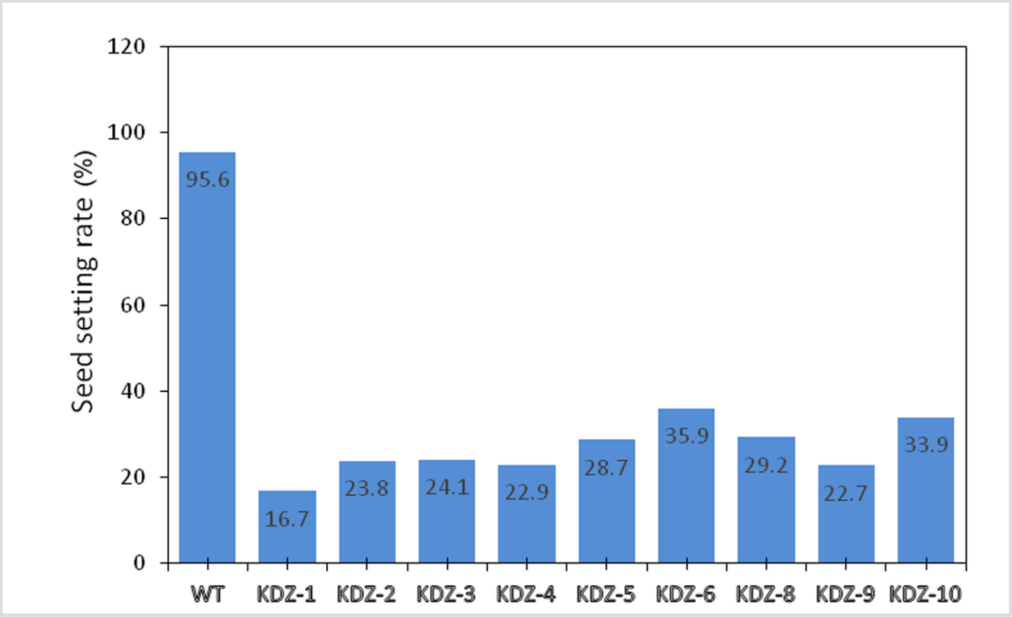

Supplementary Figure S4 Full seed percentages of primary *OsZOU-1* knock-down plants. The total number of fully-filled seeds divided by the total number of seeds represents the full seed percentage.

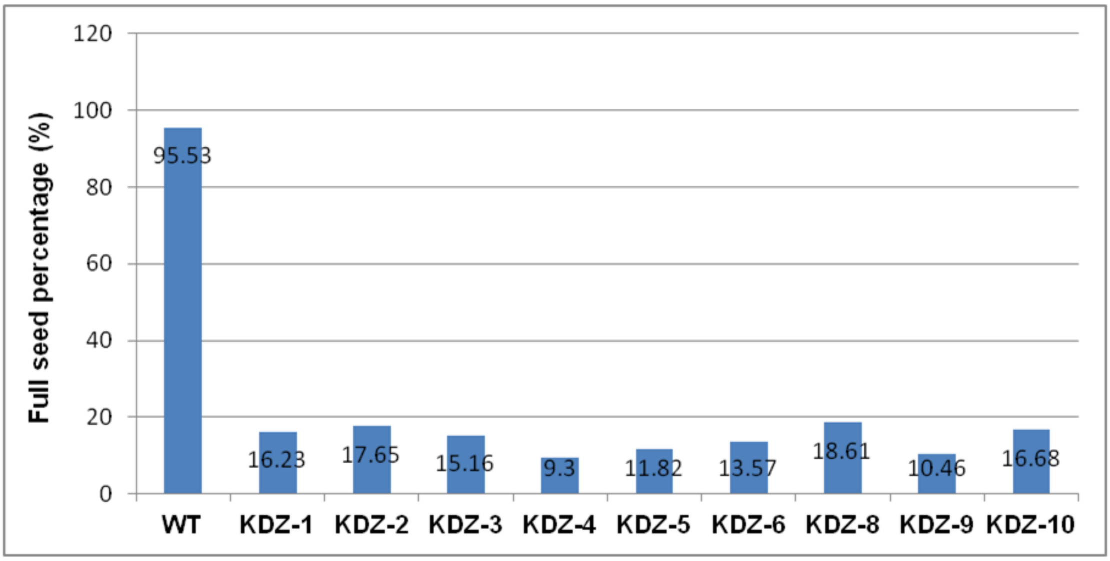

Supplementary Figure S5 Original images for Figure 5A.

(a) The left was WT and the right was KDZ-2.

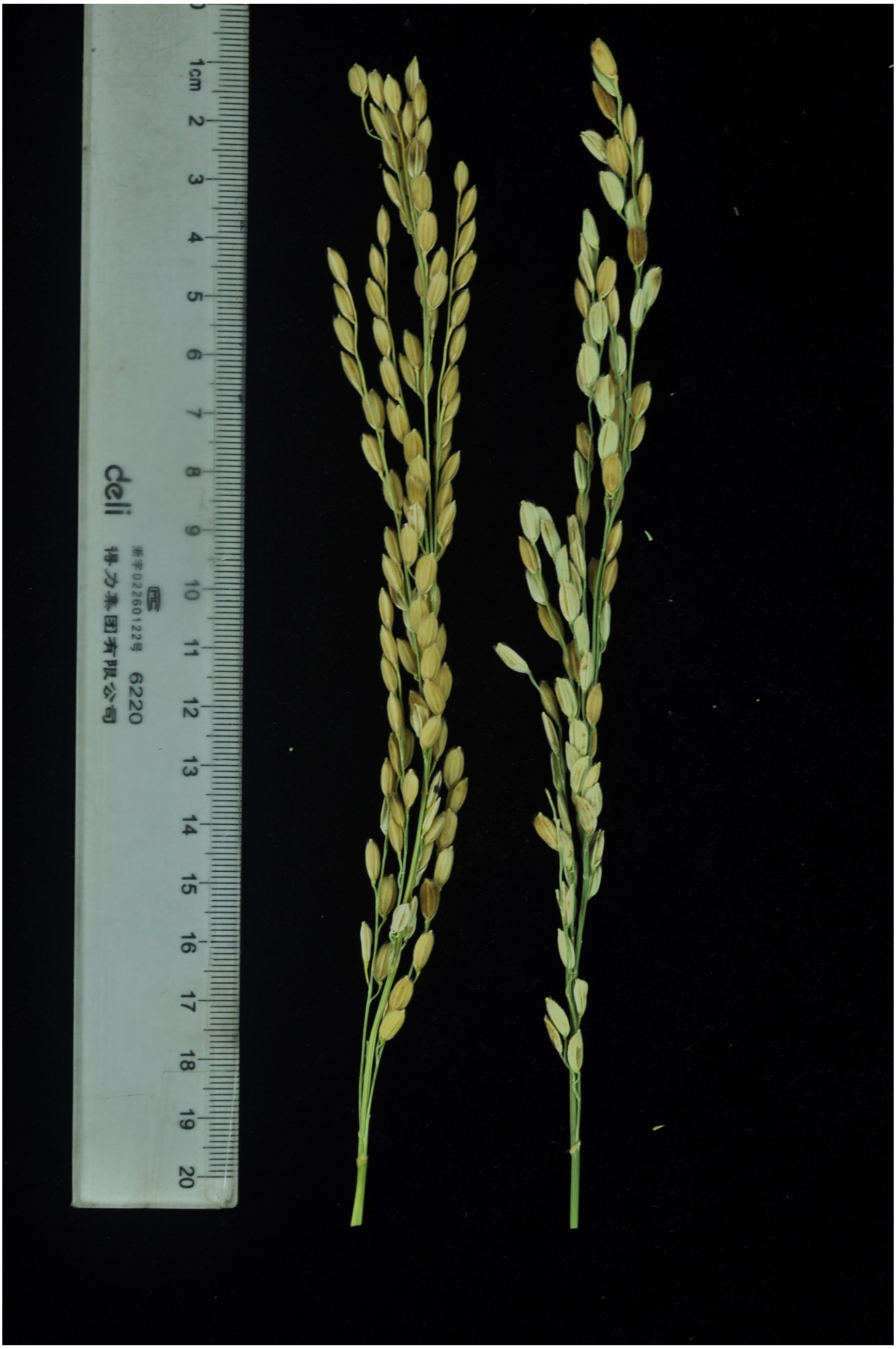

(b) The left was WT and the right was KDZ-4.

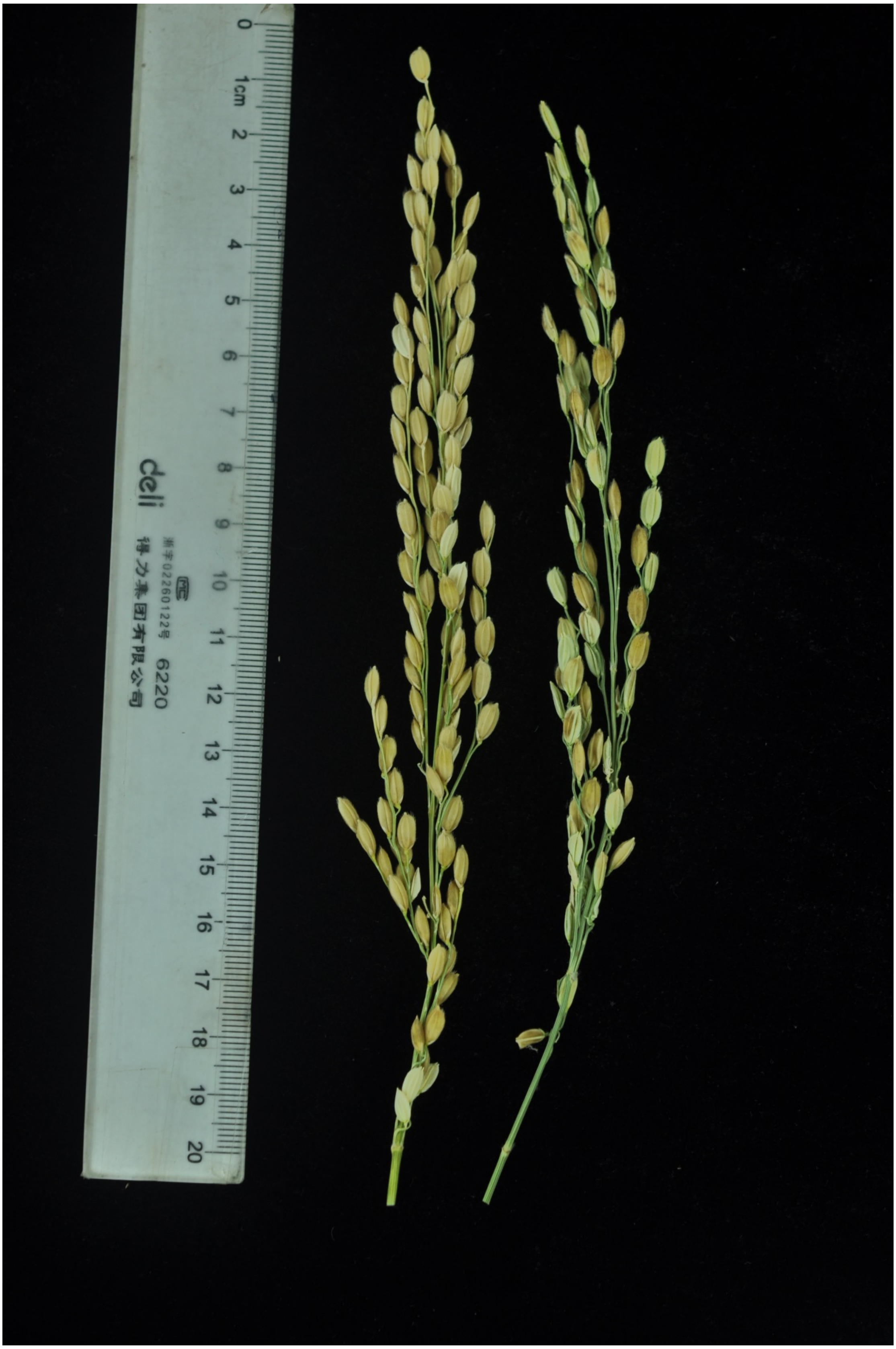

Supplementary Figure S6 Original images for Figure 5E.

(a) The seedlings of WT plant on the third day after germination were stained with TB.

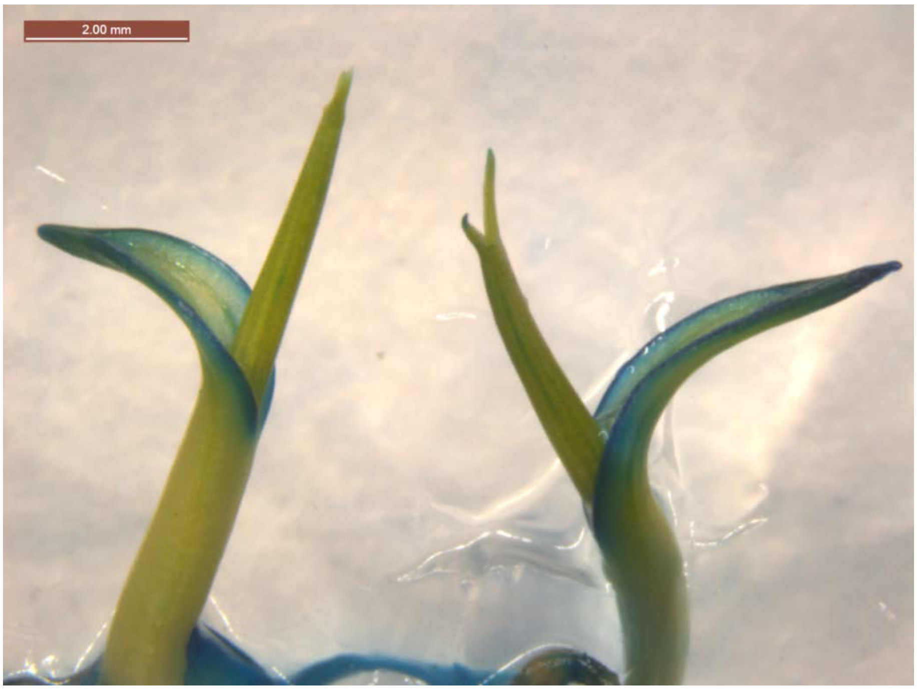

(b) The seedlings of the KDZ lines on the third day after germination were stained with TB. The left seedling was from the KDZ-1 line and the right seedling was from KDZ-2 line.

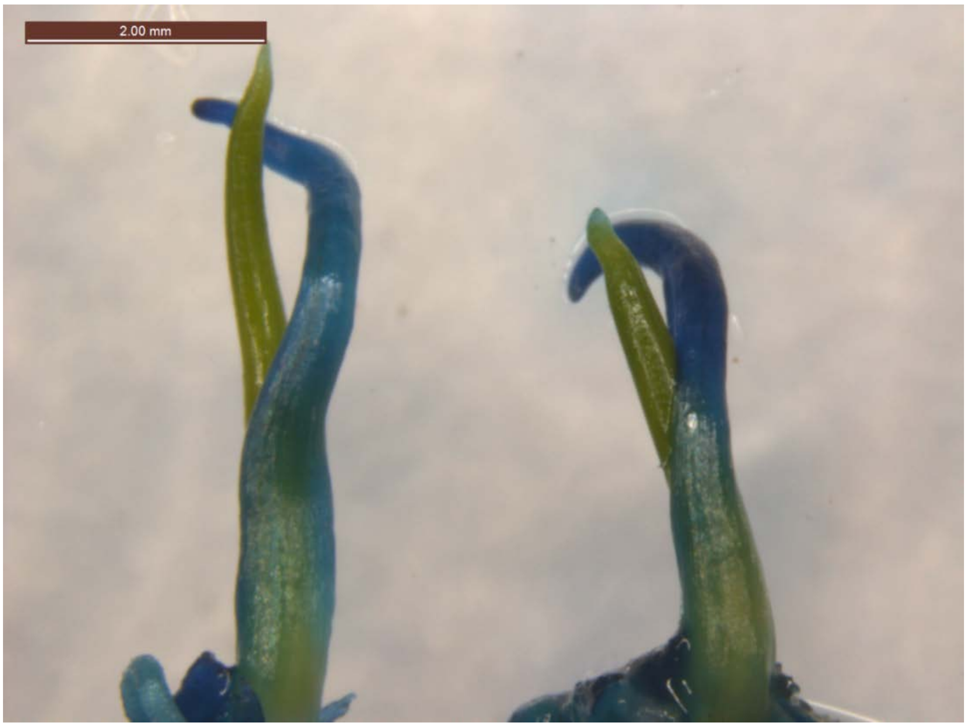

(c) The seedlings of the KDZ lines on the third day after germination were stained with TB. The left seedling was from the KDZ-1 line and the right seedling was from KDZ-4 line.

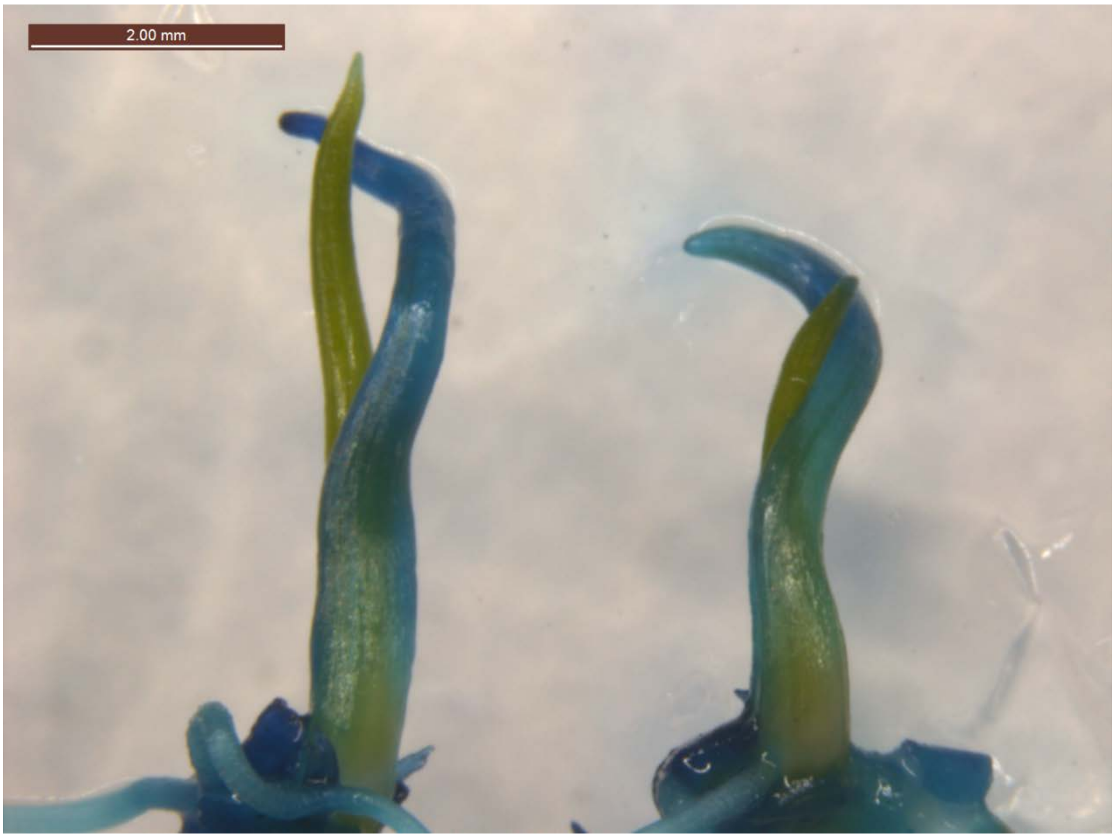

Supplementary Figure S7 Original images for Figure 6A. The plants in the same original image were not planted on the same day. To ensure that the plants used for plant height comparison were at the same stage of development, plants from different images were grouped. The WT plant in Figure 6A was the right plant of (a), its height is 81.28 cm; the OXZ-4 in Figure 6A was the right plant of (b), its height is 47.52 cm ; the OXZ-5 in Figure 6A was the left plant of (c), its height is 54.48 cm.

(a) The morphology of WT (right) and *OsZOU-1* overexpression plant OXZ-1 (left) when the WT plant was in the jointing-booting stage. The right plant was WT, because PCR result proved there was no transgene in it. The two plants were not planted on the same day.

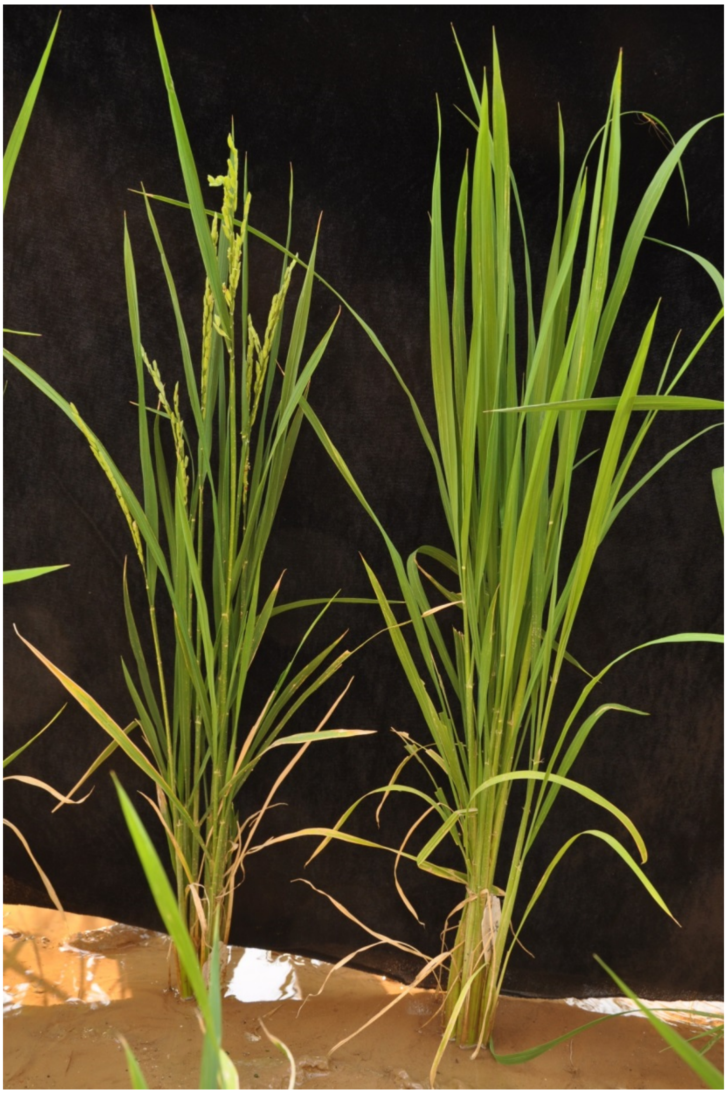

(b) The morphology of WT (left) and *OsZOU-1* overexpression plant OXZ-4 (right) when OXZ-4 was in the jointing-booting stage. The left plant was WT, because PCR result proved there was no transgene in it. The right plant was OXZ-4. The two plants were not planted on the same day.

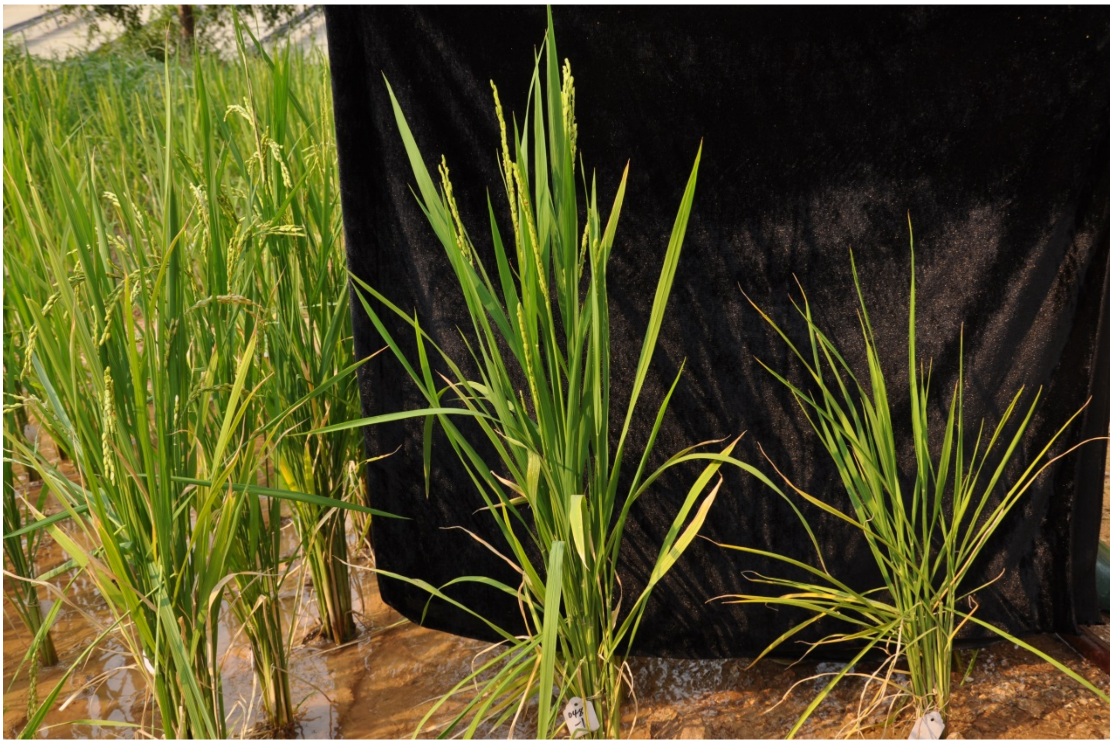

(c) The morphology of WT (right) and *OsZOU-1* overexpression plant OXZ-5 (left) when OXZ-5 was in the jointing-booting stage. The right plant was WT, because PCR result proved there was no transgene in it. The two plants were not grown on the same day.

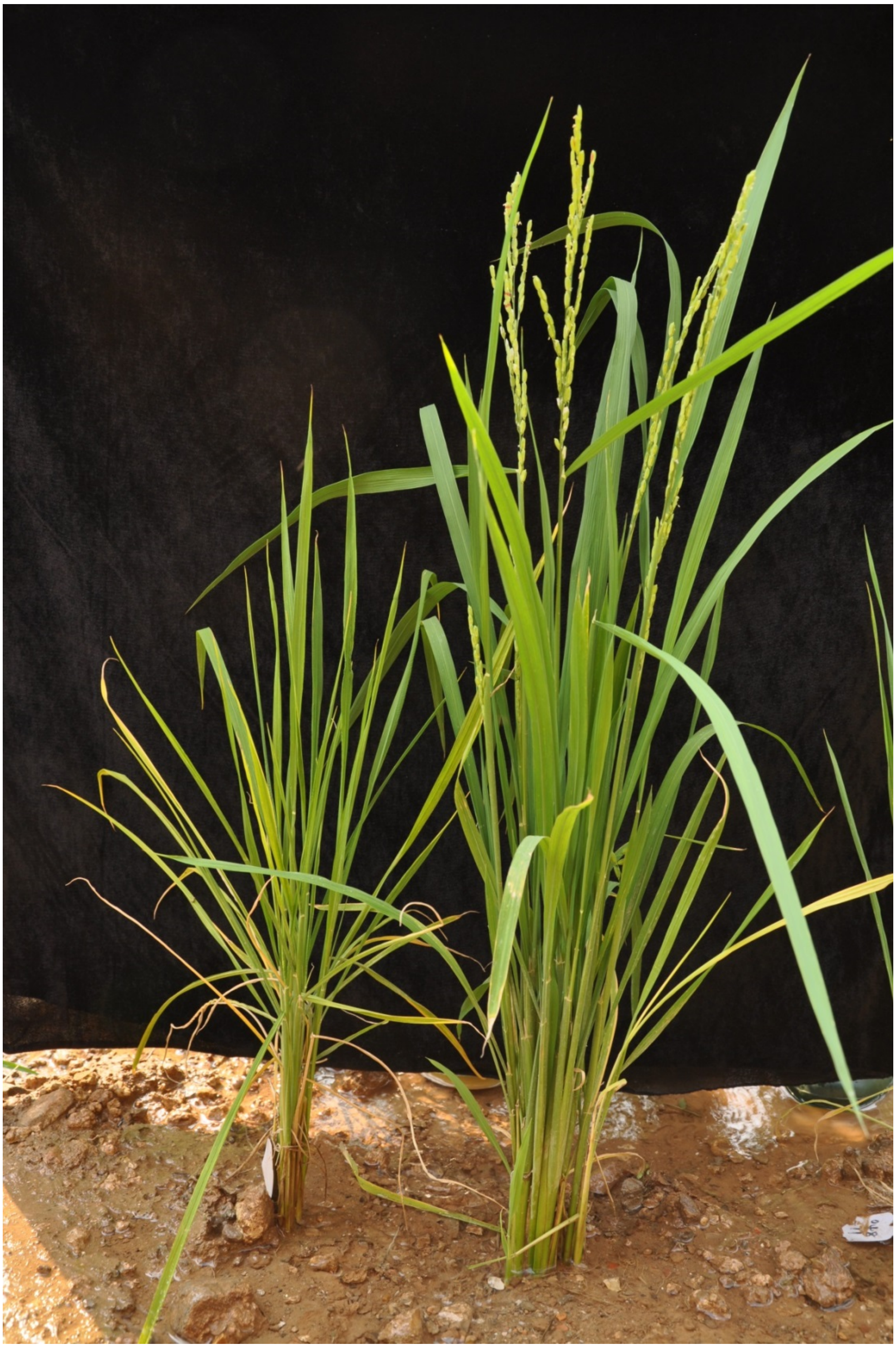

Supplementary Figure S8 Original images for Figure 6C.

(a) The left was WT and the right was OXZ-5.

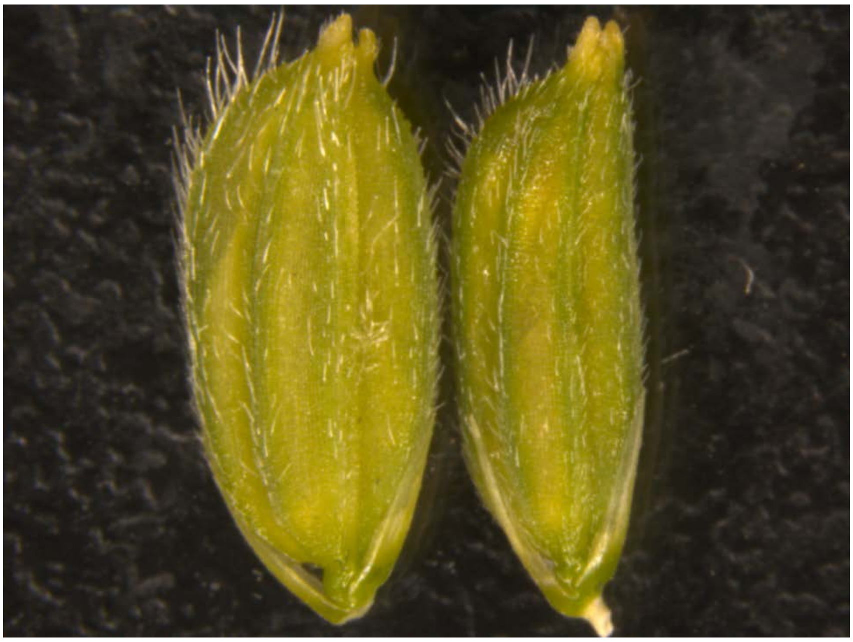

(b) The left was WT and the right was OXZ-4.

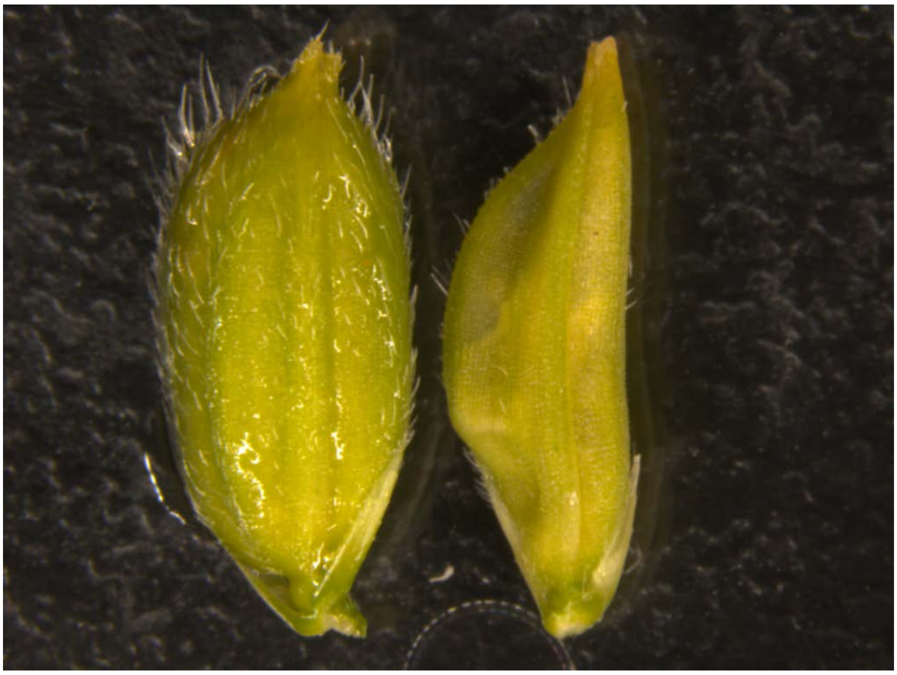

(c) The left and the right were WT and the middle was OXZ-5.

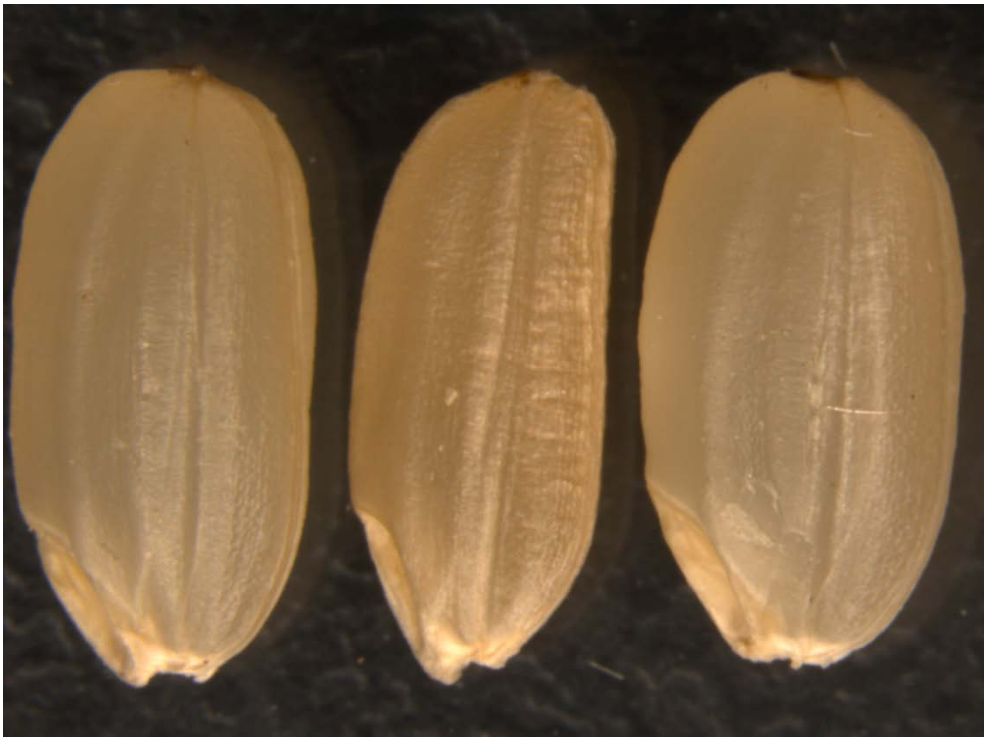

Supplementary Figure S9 Original images for Figure 6F.

(a) The germination situation of WT seed that was put in water 6 days ago.

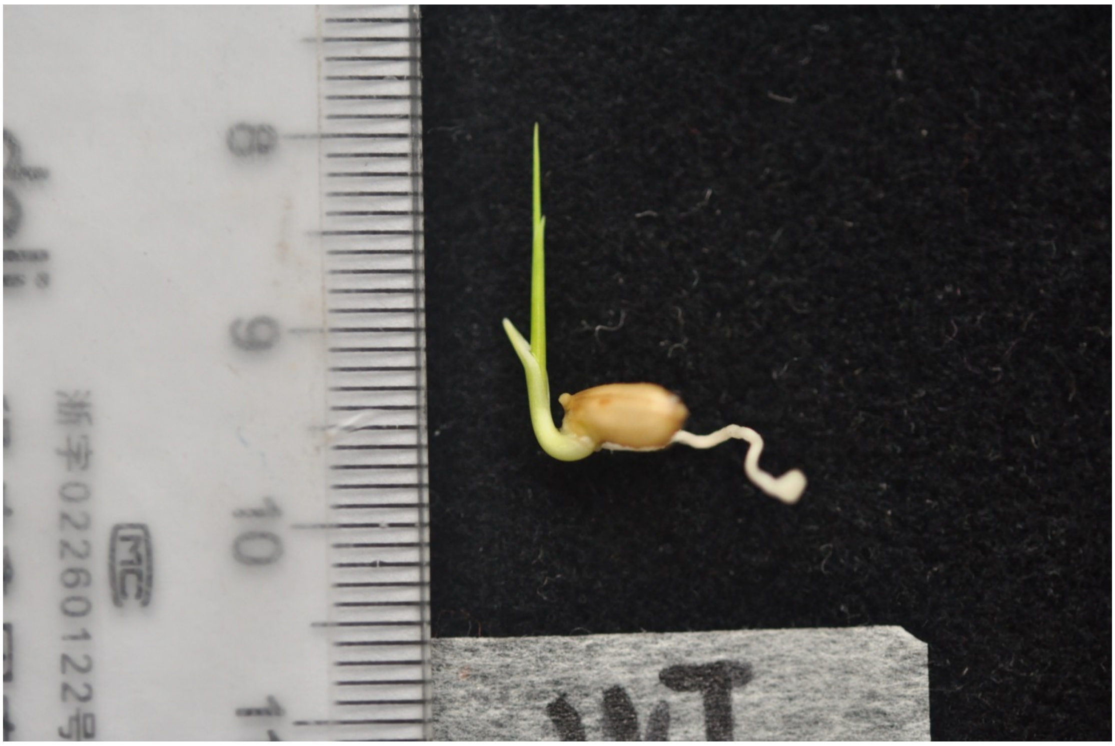

(b) The germination situation of OXZ-5 seeds that were put in water 6 days ago.

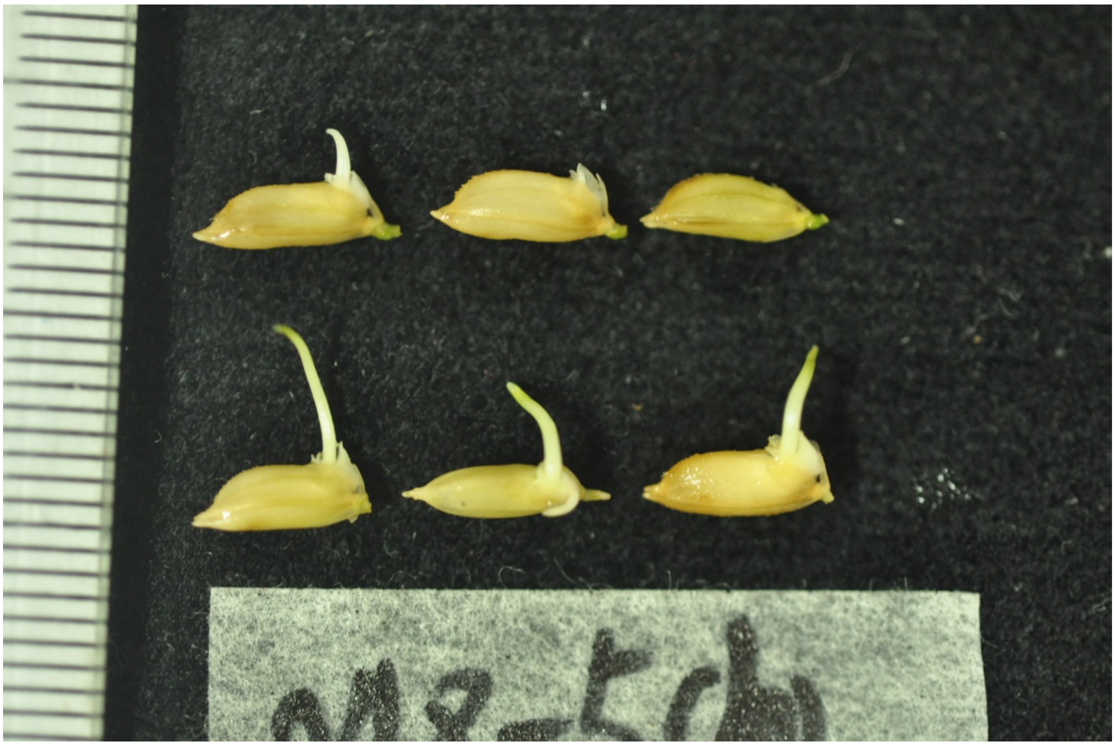

Supplemental Table 1 Primers used in this paper.

ID	Sequence	Note
OL0069	5' -CCCTCCTCCCCAATTCCCAATCTC -3'	OsZOU-1 gene clone (Forward)
OL0070	5'-GGGGGACAGCAAATTCAGGACGATC -3'	OsZOU-1 gene clone (Reverse)
OL0071	5' -ATGGCGCAAGAGGGCACGAG -3'	OsZOU-2 gene clone (Forward)
OL0072	5'-TCACATTATCTCCGAGACAGCCAG- 3'	OsZOU-2 gene clone (Reverse)
OL6076	5'- GTGGACGCGTTCATCAACAT -3'	OsZOU-1 Q-PCR (Forward)
OL6077	5'- GATGCTTCGTTGAGGCGGA -3'	OsZOU-1 Q-PCR (Reverse)
OL6078	5'- GAGCGGCGGAGGAAGATC -3'	OsZOU-2 Q-PCR (Forward)
OL6079	5'- GATGGCCTCCCCGACGAT -3'	OsZOU-2 Q-PCR (Reverse)
OL2941	5'- ATCCTTGTATGCTAGCGGTCGA -3'	OsACT1 reference gene (Forward)
OL2942	5'-ATCCAACCGGAGGATAGCATG -3'	OsACT1 reference gene (Reverse)
ZOUSALIF	5'-GTCGACTGTGGTGGCATAATACGA-3'	AtZOU Promoter clone (Forward)
ZOUSALIR	5'-GTCGACTGCTCATTTTACCCTTTT-3'	AtZOU Promoter clone (Reverse)
ZOUECORIF	5'-TGAATTCGGACAGAGAGAGTAAGAT CAGGA-3'	OsZOU-1 Promoter clone (Forward)
ZOUECORIR	5'-CCTCCTCTAGTCCCAACACCTCCA-3'	OsZOU-1 Promoter clone (Reverse)
OL3809	5'-TCGCCAGAGATCCAGACTACGACG-3'	AtZOUPI Q-PCR (Forward)
OL3795	5'-CAAGGGATTTGATTGAACTCACTGC-3'	AtZOUPI Q-PCR (Reverse)
OL2598	5'-TCTCTCGCAATCTTCGCTCTTCTC-3'	AtEIF4A1 reference gene (Forward)
OL2599	5'-GTTTCTGGTCAAACTGACGTGCATC-3'	AtEIF4A1 reference gene (Reverse)
